# Supplementary material for: Phytocystatin 6 is a context‐dependent, tight‐binding inhibitor of Arabidopsis thaliana legumain isoform β
Source: Plant J. 2023 Sep 9;116(6):1681–95. doi: 10.1111/tpj.16458 (PMC10952133; doi:10.1111/tpj.16458)
Supplement: Supplementary file 1 — Figure S1. Sequence alignment of the seven predicted phycys isoforms of A. thaliana. Figure S2. AlphaFold prediction of AtCYT6 suggested two cystatin‐like domains separated by an inter‐domain linker. Figure S3. The K i of indicated AtCYT6 constructs towards AtLEGβ and papain was determined using Morrison's equation. Figure S4. Western‐blot experiments showed that AtCYT6 was processed in A. thaliana seed extract. Figure S5. Models of AtLEGβ in complex with AtCYT6‐FL, AtCYT6‐NTD, or ‐CTD. Figure S6. Sequence alignment of five type II phycys with demonstrated inhibitory activity against PLCPs and legumains. Figure S7. Thermal stability of different AtCYT6 constructs. Figure S8. Inhibition assays of AtCYT6 constructs against AtLEGγ and hLEG. Figure S9. Structure alignment of the modeled AtCYT6‐CTD with human cystatin C. Table S1. Mass spectrometry analysis (intact mass) of AtCYT6‐derived constructs expressed in E. coli. Table S2. Cleavage sites (P1 residues) of AtLEGβ within AtCYT6‐NTD and AtCYT6‐CTD identified by mass spectrometry analysis. [file TPJ-116-1681-s001.pdf]

SUPPORTING INFORMATION

Phytocystatin 6 is a context-dependent, tight-binding inhibitor of *Arabidopsis thaliana* legumain isoform β

Naiá P. Santos[1], Wai Tuck Soh[1,6], Fatih Demir[2,7], Raimund Tenhaken[3], Peter Briza[1], Pitter F. Huesgen[2,4,5], Hans Brandstetter[1], Elfriede Dall[1,*]

Affiliations:

[1]Department of Biosciences and Medical Biology, University of Salzburg, 5020 Salzburg, Austria.

[2]Central Institute for Engineering, Electronics and Analytics, ZEA-3, Forschungszentrum Jülich, 52428 Jülich, Germany.

[3]Department of Environment and Biodiversity, University of Salzburg, 5020 Salzburg, Austria.

[4]CECAD, Medical Faculty and University Hospital, University of Cologne, 50931 Cologne, Germany

[5]Institute for Biochemistry, Faculty of Mathematics and Natural Sciences, University of Cologne, 50674 Cologne, Germany

[6]current address: Max Planck Institute for Multidisciplinary Sciences, D-37077 Göttingen, Germany

[7]current address: Department of Biomedicine, Aarhus University, 8000 Aarhus C, Denmark

Corresponding Author:

[*]Elfriede Dall (elfriede.dall@plus.ac.at)

Supplementary Figures

```
AtCYT-2      -------MATM--LKVSLVLSLLGFLVIAVVTPSAANPFRKSVVLGGKSGVPNIRTN-RE    50
AtCYT-7      MDMRRASMCMMLICVSLVLLSGFGQFVICSEEKGTYNDNVVKMKLGGFSDSKNDWNGGKE    60
AtCYT-1      --------------------------------MAD---QQAGTIVGGVRDIDANAND-LQ    24
AtCYT-3      --MESKT-----FWIVTLLLCGTIQLAICRSEEKS---TEKTMMIGGVHDLRGNQNS-GE    49
AtCYT-6      -MMRSRFLLFIVFFSLSLFISS----LIASDLGFC---NEEMALVGGVGDVPANQNS-GE    51
AtCYT-4      ------MMMKSLICLSLILLPLVSVV----------EGLGGGGGIGSRKPIKNV--SDPD    42
AtCYT-5      --MTSKVVFLLLLSLVVVLLPLYA---------------SAAARVGGWSPISNV--TDPQ    41
                                                         :*.            :

AtCYT-2      IQQLGRYCVEQFNQQAQNEQGNIGSIAKTDTAISNPLQFSRVVSAQKQVVAGLKYYLRIE    110
AtCYT-7      IDDIALFAVQEHNRRE-----------------NAVLELARVLKATEQVVAGKLYRLTLE    103
AtCYT-1      VESLARFAVDEHNKNE-----------------NLTLEYKRLLGAKTQVVAGTMHHLTVE    67
AtCYT-3      IESLARFAIQEHNKQQ-----------------NKILEFKKIVKAREQVVAGTMYHLTLE    92
AtCYT-6      VESLARFAVDEHNKKE-----------------NALLEFARVVKAKEQVVAGTLHHLTLE    94
AtCYT-4      VVAVAKYAIEEHNKES-----------------KEKLVFVKVVEGTTQVVSGTKYDLKIA    85
AtCYT-5      VVEIGEFAVSEYNKRS-----------------ESGLKFETVVSGETQVVSGTNYRLKVA    84
             :  :. :.:.:.*:.                  .  *    :: .  ***:*  : * :

AtCYT-2      VTQPNGSTRMFDSVVVIQPWLHSKQLLGFTPVVSPVY-----------------------    147
AtCYT-7      VIEAG-EKKIYEAKVWVKPWMNFKQLQEFKNI--IPSFTISDLGFKPDGNGFDWRSVSTN    160
AtCYT-1      VADGE-TNKVYEAKVLEKAWENLKQLESFNHLHDV-------------------------    101
AtCYT-3      AKEGD-QTKNFEAKVWVKPWMNFKQLQEFKESSS--------------------------    125
AtCYT-6      ILEAG-QKKLYEAKVWVKPWLNFKELQEFKPASDAPAITSSDLGCKQGEHESGWREVPGD    153
AtCYT-4      AKDGGGKIKNYEAVVVEKLWLHSKSLESFKAL----------------------------    117
AtCYT-5      ANDGDGVSKNYLAIVWDKPWMKFRNLTSFEPANNGRFL----------------------    122
               :     : : : *  : * : :.*  *

AtCYT-2      ------------------------------------------------------------    147
AtCYT-7      NPEVQEAAKHAMKSLQQKSNSLFPYKLIDIILARAKVVEERVKFELLLKLERGNKLEKFM    220
AtCYT-1      ------------------------------------------------------------    101
AtCYT-3      ------------------------------------------------------------    125
AtCYT-6      DPEVKHVAEQAVKTIQQRSNSLFPYELLEVVHAKAEVTGEAAKYNMLLKLKRGEKEEKFK    213
AtCYT-4      ------------------------------------------------------------    117
AtCYT-5      ------------------------------------------------------------    122

AtCYT-2      ---------------------    147
AtCYT-7      VEVMKDQTGKYE---------    232
AtCYT-1      ---------------------    101
AtCYT-3      ---------------------    125
AtCYT-6      VEVHKNHEGALHLNHAEQHHD    234
AtCYT-4      ---------------------    117
AtCYT-5      ---------------------    122
```

Supplementary figure 1. Sequence alignment of the seven predicted phycys isoforms of *A. thaliana*. Sequences were retrieved from Uniprot entries Q945Q1 (AtCYT1), Q8L5T9 (AtCYT2), Q41906 (AtCYT3), Q84WT8 (AtCYT4), Q41916 (AtCYT5), Q8H0X6 (AtCYT6) and Q8LC76 (AtCYT7). Motifs for inhibitory activity against papain and legumain are highlighted in solid black boxes and dashed black box, respectively.

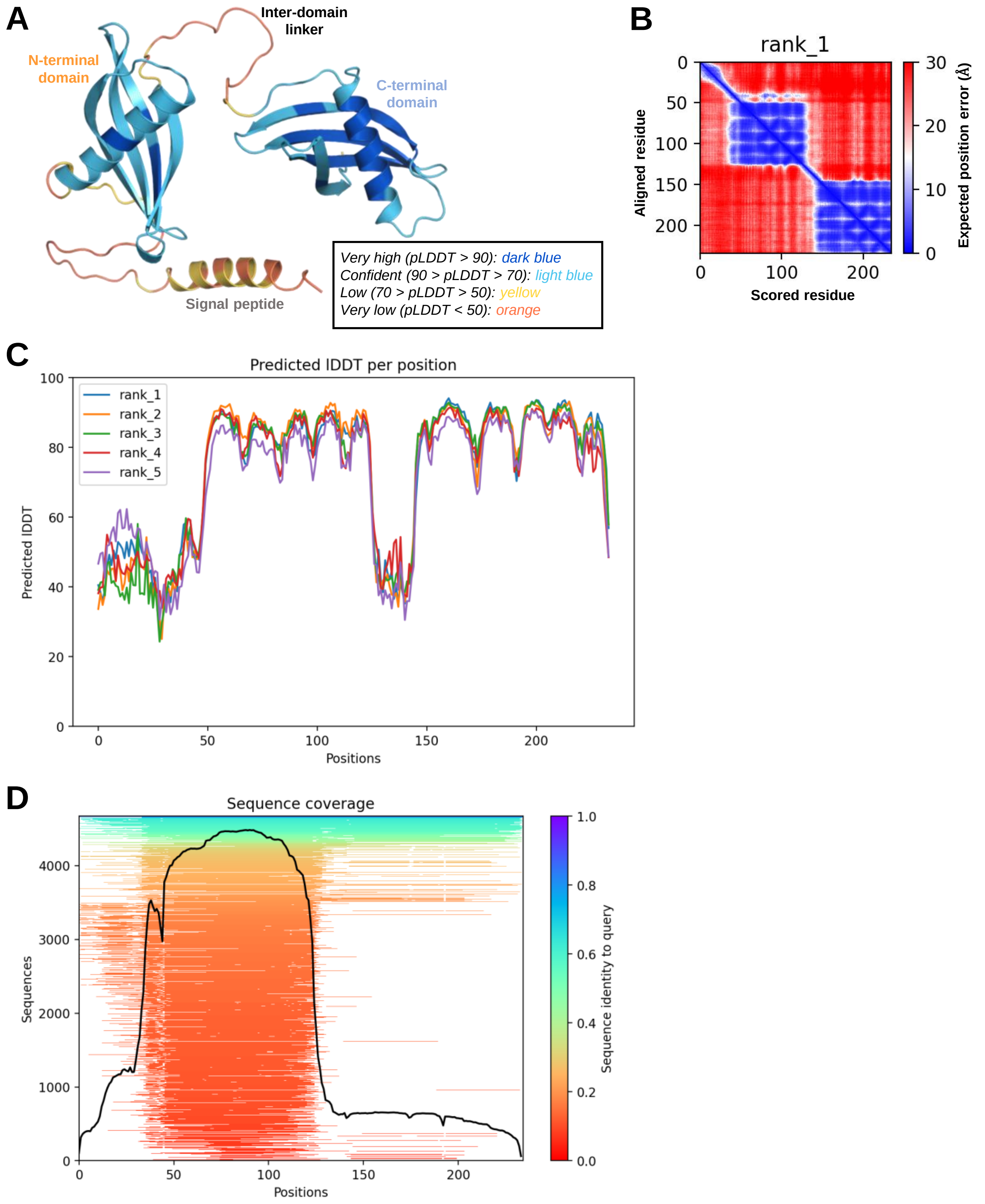

Supplementary figure 2. AlphaFold prediction of AtCYT6 suggested two cystatin-like domains separated by an inter-domain linker. (A) AlphaFold model of AtCYT6 in ribbon representation, colour-coded according to the per-residue predicted local distance difference test (pLDDT) value. The AlphaFold run resulted in 5 separate models. The model with the highest overall pLDDT (pLDDT: 74) is shown (rank_1). **(B)** Plot showing the predicted aligned error, which revealed that there is no interaction between the cystatin domains. **(C)** Per-residue pLDDT plot for the 5 models generated by AlphaFold. **(D)** The sequence coverage plot revealed a low number of sequences with high sequence identity and in general less homologous sequences to the C-terminal cystatin domain as compared to the N-terminal domain.

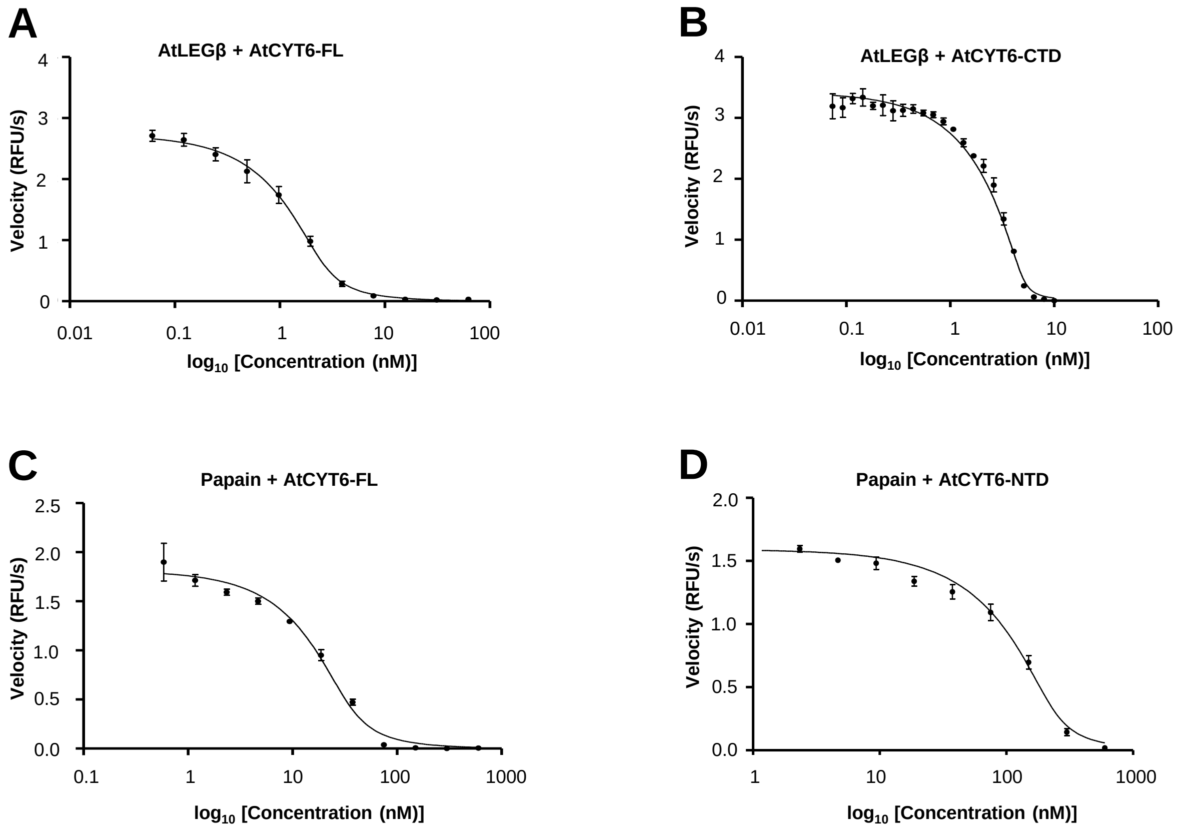

Supplementary figure 3. The K_i of indicated AtCYT6 constructs towards AtLEGβ and papain was determined using Morrison's equation. (A) and **(B)** Activity was measured at pH 5.5 using the AAN-AMC substrate. **(C)** and **(D)** Activity was measured at pH 6.5 using the FR-AMC substrate.

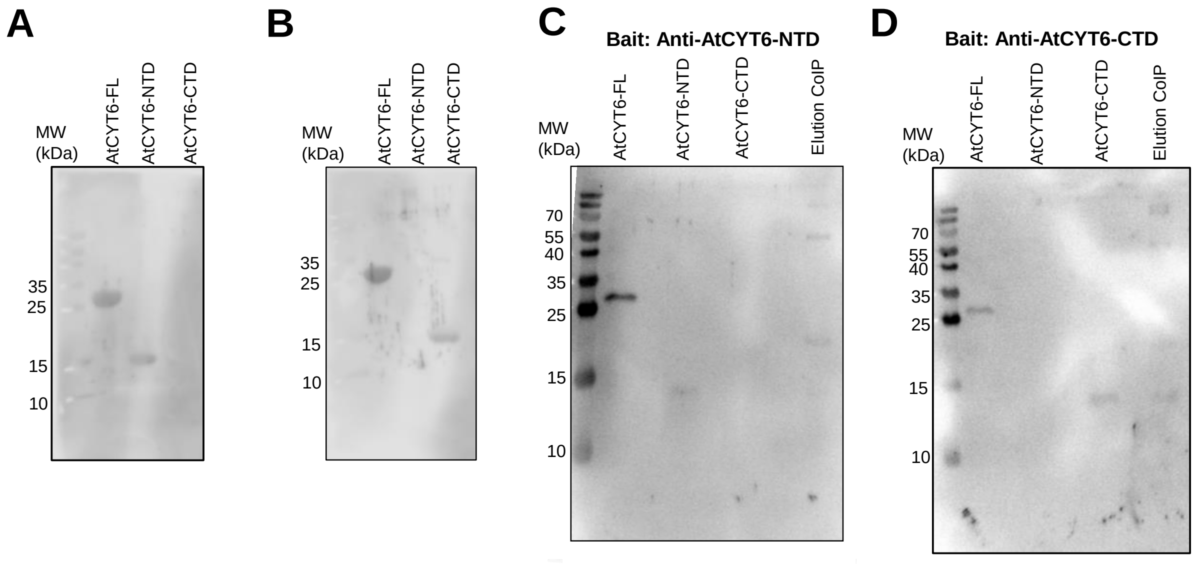

Supplementary figure 4. Western-blot experiments showed that AtCYT6 was processed in *A. thaliana* seed extract. (A) Western blot against indicated recombinant AtCYT6 proteins using anti-AtCYT6-NTD antiserum as primary antibody. **(B)** Same as **(A)** but using anti-AtCYT6-CTD antiserum. **(C)** A seed extract was prepared at pH 5.5 and co-immunoprecipitated with immobilized anti-AtCYT6-NTD antibody as a bait. The elution fraction was analyzed by western blotting using anti-AtCYT6-NTD antibodies as primary antibody. **(D)** Same as **(C)** but using anti-AtCYT6-CTD as bait in the CoIP and anti-AtCYT6-CTD antibodies for western blotting. Indicated recombinant AtCYT6 proteins were loaded for size comparison. The elutions of the CoIPs displayed bands corresponding in size to the individual AtCYT6-NTD or –CTD respectively.

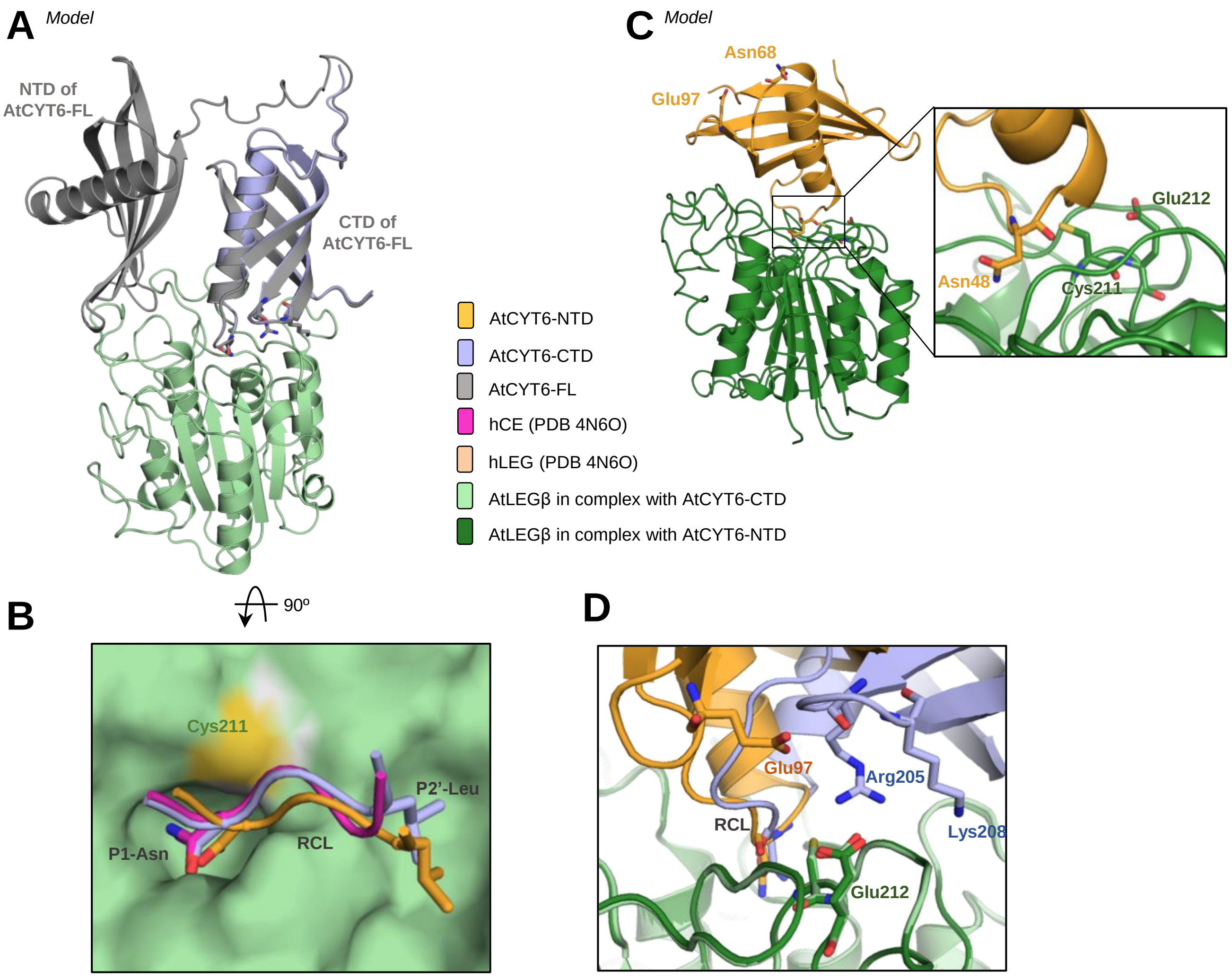

Supplementary figure 5. Models of AtLEGβ in complex with AtCYT6-FL, AtCYT6-NTD or -CTD. (A) Binding mode of AtCYT6-FL (grey) to AtLEGβ as predicted by AlphaFold Multimer. The CTD of the full-length protein binds to AtLEGβ (green) with the same orientation as AtCYT6-CTD (blue) and does not interact with AtCYT6-NTD. **(B)** Top-view on the RCL bound to the active site of AtLEGβ (green surface). Orange: AtCYT6-NTD, blue: AtCYT6-CTD, pink: hCE. **(C)** Binding mode of AtCYT6-NTD to AtLEGβ as originally predicted by AlphaFold Multimer. In the model, the AtCYT6-NTD (wheat) binds to AtLEGβ (green) with the Asn48 inserted into the active site, whereas Asn68 and Glu97 are positioned on the opposite side of the active site of AtLEGβ. **(D)** Zoom-in view on the LEL. In the model, residues Glu97 of AtCYT6-NTD (orange), and Arg205 and Lys208 of AtCYT6-CTD (blue) were in close proximity to Glu212 on AtLEGβ (green).

```
FcCYT1      MKLKS----QS----NSSPSFVVFFVLFFSF--------------IALSEMSTLGGIRDS 38
FaCYT1      MKLRS----QS----NSSPSFVFFFVLFFSF--------------IALSEMATLGGIRDS 38
AtCYT6      ---------------MMRSRFLLF-IVFFSL-SLFISSLIASDLGFCNEEMALVGGVGDV 43
OsCYT12     MRVAATTRPASSSAAAPLPLFLLL-AVAAAAAALFLVG--SASLA---MAGHVLGGAHDA 54
HvCYT4      MRVAAT-RPVSSA---PV-------ALLAALALLFLVG--SASLAIGTMASHVLGGKSEN 47
                                      :  :                       :**  :

FcCYT1      PAGSENSLETEALGRFAVDDHNQKQNGMLEFVRVVKAKEQVVSGTLHHLVVEAIDGGKKK 98
FaCYT1      PAGSENSLETEALGRFAVDDHNQKQNGMLEFVRVVKAKEQVVAGTLHHLVVEAIDGGKKK 98
AtCYT6      PA-NQNSGEVESLARFAVDEHNKKENALLEFARVVKAKEQVVAGTLHHLTLEILEAGQKK 102
OsCYT12     PS-AANSVETDALARFAVDEHNKRENALLEFVRVVEAKEQVVAGTLHHLTLEALEAGRKK 113
HvCYT4      PA-AANSLETDGLARFAVDEHNKRENALLEFVRVVEAKEQTVAGTLHHLTLEALEAGRKK 106
            *:   ** *.:.*.*****:**:::*.:***.***:****.*:******.:* ::.*:**

FcCYT1      LYEAKVWVKPWLNFKQVQEFKHAGDPETVSGTPSFTSSDLGVKQGGHAPGWQDVHPHDPQ 158
FaCYT1      LYEAKVWVKPWLNFKQVQEFKHAGEPETVSGTPSFTSSDLGVKQGGHAPGWQDVHPQDPQ 158
AtCYT6      LYEAKVWVKPWLNFKELQEFKPASDA------PAITSSDLGCKQGEHESGWREVPGDDPE 156
OsCYT12     VYEAKVWVKPWLDFKELQEFRNTGDA------TTFTNADLGAKKGGHEPGWRDVPVHDPV 167
HvCYT4      VYEAKVWVKPWLDSKELQEFRHTGDA------TSFTISDLGAKRGGHEPGWRDVPVHDPV 160
            :***********: *::***: :.:        ::* :*** *:* *  **::*  .**

FcCYT1      VQDAANHAVKSLQQKSNSLFPYELQEVVHAKAEVMEEHAKFNMLLKLKRGDKEEKYKVEV 218
FaCYT1      VQDAANHAVKSLQQKSNSLFPYELQEVVHAKSEVMEEHAKFNMLLKLKRGDKEEKYKVEV 218
AtCYT6      VKHVAEQAVKTIQQRSNSLFPYELLEVVHAKAEVTGEAAKYNMLLKLKRGEKEEKFKVEV 216
OsCYT12     VKDAADHAVKSIQQRSNSLFPYELLEIVRAKAEVVEDFAKFDILMKLKRGNKEEKFKAEV 227
HvCYT4      VKDAASHAVKSIQERSNSLFPYELIEIVRAKAEVVEDFAKFDIVMKLKRGTKEEKMKAEV 220
            *:..*.:***::*::********* *:*:**:**  : **:::::***** **** *.**

FcCYT1      HKNNEGAYNLNQMEVEH------ 235
FaCYT1      HKNNEGAYNLNQMEVEH------ 235
AtCYT6      HKNHEGALHLNHAEQHHD----- 234
OsCYT12     HKNLEGAFVLNQMQQEHDESSSQ 250
HvCYT4      HKNLEGAFVLNQMQPEHDESSSQ 243
            *** ***  **: : .*
```

Supplementary figure 6. Sequence alignment of five type II phycys with demonstrated inhibitory activity against PLCPs and legumains. Sequences were retrieved from Uniprot entries Q0JNR2 (OsCYT12, *Oryza sativa* cystatin 12), Q1ENE9 (HvCYT4, *Hordeum vulgare* cystatin 4), Q8H0X6 (AtCYT6, *A. thaliana* cystatin 6), Q4GZT8 (FaCYT1, *Fragaria ananassa* cystatin 1) and A0A2U9AEI9 (FcCYT1, *Fragaria chiloensis* cystatin 1). The P1-Asn residue on the RCL of the N- and C-terminal domains is indicated by solid and dashed black boxes, respectively. The conserved R-G-X-K double-basic motif on the LEL of the legumain-inhibitory CTDs is highlighted by a dashed grey box, and aspartate and glutamate residues on the LEL of the N-terminal domains by a solid grey box.

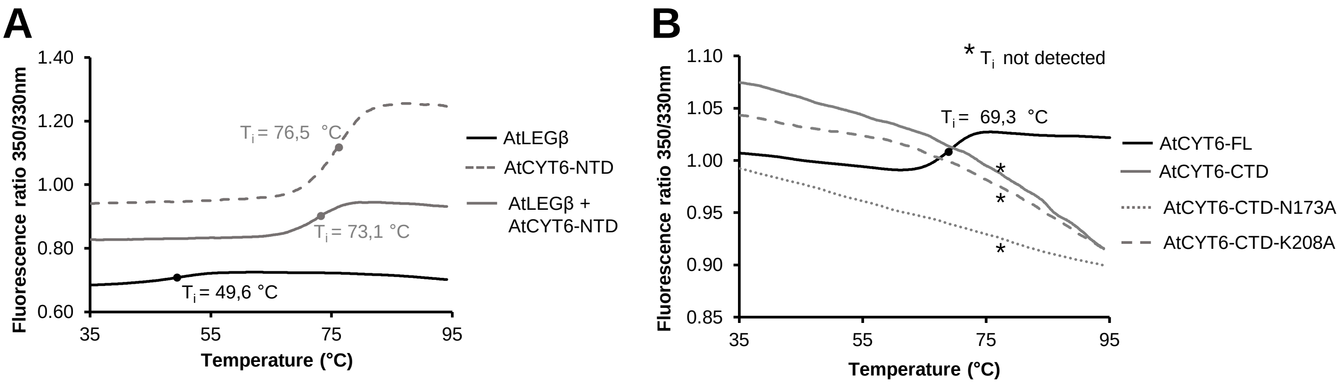

Supplementary figure 7. Thermal stability of different AtCYT6 constructs. The thermal stability of AtLEGβ and AtCYT6 constructs was measured by nanoDSF (differential scanning fluorimetry) measurements. Unfolding transitions (inflection point; T_i) are indicated by solid circles. **(A)** AtLEGβ was inhibited with MMTS and incubated at pH 6.5 in the absence (black) and presence of AtCYT6-NTD (dark grey). AtCYT6-NTD alone is shown as dashed grey line. **(B)** Denaturation curves obtained for AtCYT6-FL (solid black line), AtCYT6-CTD (grey solid line) AtCYT6-CTD-N173A (grey dotted line) and AtCYT6-CTD-K208A (grey dashed line) at pH 6.5 as controls for experiments depicted in Fig. 7.

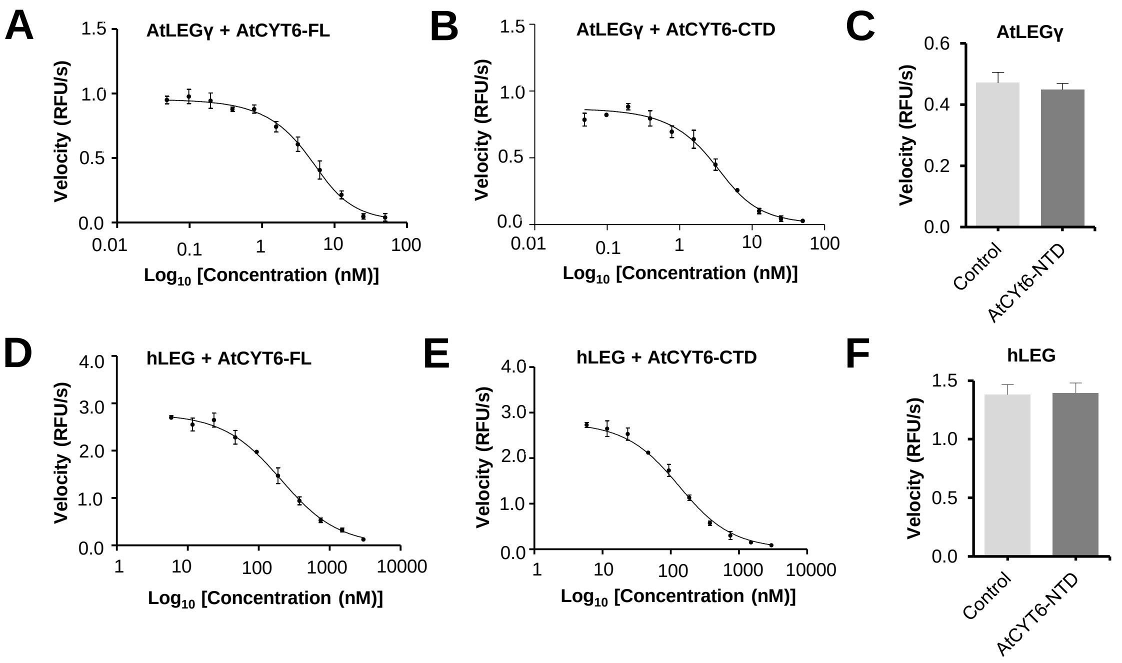

Supplementary figure 8. Inhibition assays of AtCYT6 constructs against AtLEGγ and hLEG. Enzymatic activity was measured at pH 5.5 as the increase in fluorescence after turnover of the AAN-AMC substrate. **(A)** and **(B)** The Ki of indicated AtCYT6 constructs towards AtLEGγ was determined using Morrison's equation. **(C)** Activity of AtLEGγ in the presence (dark grey) and absence (light grey) of AtCYT6-NTD. **(D)** and **(E)** The Ki of indicated AtCYT6 constructs towards hLEG was determined using Morrison's equation. **(F)** Activity of hLEG in the presence (dark grey) and absence (light grey) of AtCYT6-NTD.

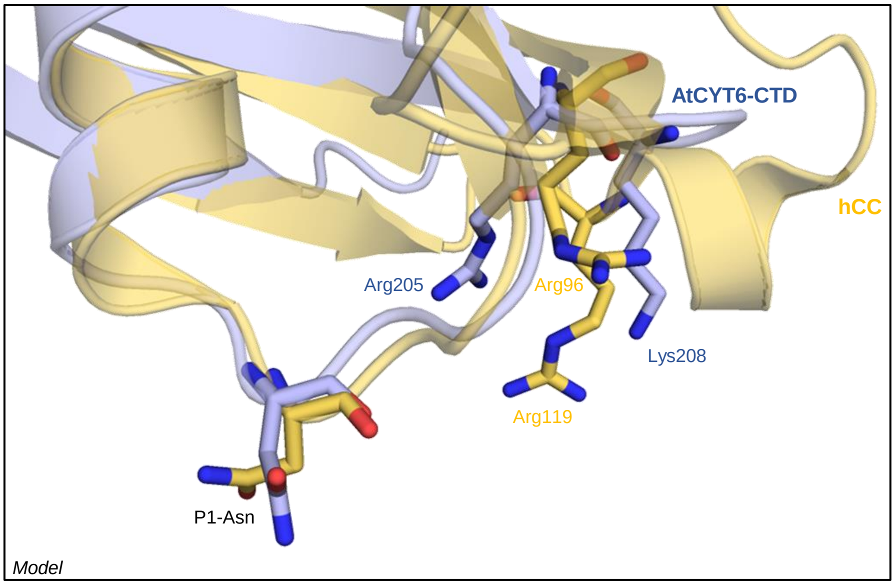

Supplementary figure 9. Structure alignment of the modeled AtCYT6-CTD (blue) with human cystatin C (hCC, PDB 3GAX; yellow). The P1-Asn residues and residues on the LEL are shown as sticks.

Supplementary Tables

Supplementary Table 1. Mass spectrometry analysis (intact mass) of AtCYT6-derived constructs expressed in *E. coli*.

Construct **(AtCYT6-)**	*Theoretical mass (Da)*	*Experimental mass (Da)*	*Delta mass (Da)*	*Position*	*Sequence*	*Relative abundance (%)*
**FL**	-	no signal	-	-	-	-
**NTD**	12611.46	12611.59	-0.13	2-115	(M)GSSH…PASD	100
**CTD**	12465.169	12465.30	-0.13	7-115	(H)HHH…QHHD	100
**CTD$_{long}$**	14299.01	14299.19	-0.18	2-129	(M)GSSH…HHD	100

Supplementary Table 2. Cleavage sites (P1 residues) of AtLEGβ within AtCYT6-NTD and AtCYT6-CTD identified by mass spectrometry analysis.

	**AtCYT6-NTD**	**AtCYT6-CTD**
**Cleavage sites**	Asn46<br>Asn68<br>Asn115	Asn173
